# An optimized substitution box generator based on cubic pell curves and its application in image encryption

**DOI:** 10.1038/s41598-025-28045-y

**Published:** 2026-01-28

**Authors:** Mohammad Abdul Mujeeb Khan, Naveed Ahmed Azam, Hailiza Kamarulhaili

**Affiliations:** 1 Department of Mathematics and Statistics, College of Arts and Sciences, Qatar University, Doha, 2713 Qatar; 2 Department of Mathematics, Quaid-i-Azam University, Islamabad, 45320 Pakistan; 3 School of Mathematical Sciences, Universiti Sains Malaysia, Minden, 11800 Malaysia

**Keywords:** Cubic Pell curve, Substitution box, Security analysis, Image encryption, Computational science, Information technology

## Abstract

In today’s digital landscape, safeguarding confidential data from cyber threats and unauthorized breaches is more crucial than ever. A key component in modern cryptographic systems is the substitution box (S-box), which ensures data security through complex transformations. Designing S-boxes with high nonlinearity and computation efficiency is still challenging. In this paper, we propose a novel S-box generator based on cubic Pell curves. The key idea of our generator is to utilize the randomness of the points over the cubic Pell curves by using their binary strings. The optimized S-boxes are obtained by performing swapping operations on initial S-boxes that ensure high nonlinearity. Cryptographic evaluations including nonlinearity (NL), strict avalanche criterion (SAC), bit independence criterion (BIC), differential approximation probability (DAP), linear approximation probability (LAP) and algebraic complexity (AC) demonstrate the strength of our method. The proposed S-boxes achieve optimal nonlinearity (108), outperform existing schemes in speed and security, and pass NIST randomness tests. Image encryption results demonstrate the robustness of the generated S-boxes against statistical attacks, further validating their cryptographic strength.

## Introduction

Researchers have explored multiple strategies to develop highly secure S-boxes for cryptographic applications. As a fundamental element in block cipher encryption and decryption, the substitution box (S-box) plays a vital role in establishing confusion between the secret key and ciphertext. S-boxes generally fall into two categories: static and dynamic. While static S-boxes maintain a fixed structure, dynamic S-boxes can adapt in size, offering greater resistance against subkey extraction and data analysis attacks. Due to this flexibility, dynamic S-boxes are often considered more effective in strengthening cryptographic security^1^. Several studies have introduced S-box constructions based on elliptic curve cryptography (ECC), highlighting their potential in modern encryption systems. Both dynamic and optimized S-box generation techniques play a crucial role in enhancing the security and efficiency of cryptographic protocols.

Elliptic Curve Cryptography (ECC) is currently the most widely used public key cryptographic system. However, the cubic Pell curve (CPC) cryptosystem provides a more efficient alternative with higher performance. The cubic Pell curves offer several unique advantages over Elliptic curves in the construction of substitution boxes (S-boxes) for cryptographic applications. These advantages stem from their distinct algebraic properties, computational efficiency, and strong security features. The cubic Pell curves are defined by the formula $\mathscr {x}^3 + \alpha \mathscr {y}^3 + \alpha ^2\mathscr {z}^3 - 3\alpha \mathscr {x}\mathscr {y}\mathscr {z} \equiv 1 \pmod {p}$, which results in a larger group order compared to elliptic curves for the same prime field. This property enables cubic Pell curves to generate more set of points, thereby offering greater randomness and variation for the construction of the S-boxes. Despite their larger group order, arithmetic operations on cubic Pell curves such as point addition law are computationally comparable to those on elliptic curves. This ensures efficient S-box generation without compromising performance. The deterministic nature of point generation on cubic Pell curves reduces computational overhead compared to the probabilistic methods often used in some elliptic curve based S-box constructions. The points on cubic Pell curves exhibit strong pseudorandom behavior due to the curve nonlinear structure. The complexity of solving the Discrete Logarithm Problem in CPC is at least as challenging as in ECC, assuming the group orders of PEC and ECC are comparable. Unlike ECC, which is based on the algebraic properties of elliptic curve point groups, CPC cryptography operates on a different class of curves derived from the solutions of a cubic Pell equation^2^. The cubic Pell curves offer flexibility in parameter selection, such as the non-cubic residue $\alpha$ and prime *p* enabling dynamic S-box generation designed to meet specific security requirements. The cubic Pell curve cryptosystem is proposed to work on a higher order but with comparable computational complexity compared to well known elliptic curve cryptosystems. Recently^3^, presented a new public key cryptosystem founded on the arithmetic of the cubic Pell curve $\mathcal{P}\mathcal{C}_\alpha (N): x^3 + \alpha y^3 + \alpha ^2z^3 - 3\alpha xyz = 1$ over $\mathbb {Z}/ N \mathbb {Z}$, with $N=p^rq^s$as a multi prime power integer. The security of this system is based on the difficulty of factoring composite integers and the one way trapdoor function introduced by Rabin. In this unique method, arithmetic operations are carried out on a cubic Pell curve, which is exclusively known to the sender and receiver of the message. The arithmetic operations on cubic Pell curves known as the generalized Brahmagupta product can be implemented efficiently in both hardware and software, making cubic Pell curves based S-boxes practical for real world applications. Efficiency in large scale S-box generation has also been a key research focus.

In this manuscript, we used a particular cubic Pell curve over large primes to design an S-box of good cryptographic strength with respect to elliptic curve-based S-boxes. So far, there is no S-box on the cubic Pell curve in the literature. We generate the points using arithmetic of the cubic Pell curve to construct a dynamic S-box. Both hardware and software can implement cubic Pell curve operations efficiently.

### Related work

Dynamic S-box generators are developed to create S-boxes with high variability while maintaining minimal computational complexity. These generators enhance encryption performance by improving efficiency and reducing the processing overhead of encryption algorithms. Various techniques have been explored for S-box construction. Reference^4^ introduced an S-box design approach that integrates two methods. Initially, they created an S-box based on a non-permutation power function, followed by a heuristic algorithm that ensures the final S-box is bijective, mapping 8-bit inputs to 8-bit outputs. While^5^ introduced an efficient algorithm for generating random bijective S-boxes by combining chaotic maps with a composition method. This approach constructs new S-boxes through compositions of a fixed initial set, where the sequence of S-box selection is determined by chaotic sequences. Performance tests demonstrate that the resulting S-boxes exhibit strong cryptographic properties. The key advantages of this method include low computational complexity and the ability to achieve a large key space. Reference^6^ proposed an algorithm to construct an $8\times 8$ S-box by applying a fractional linear transformation over the Galois field $GF(2^8)$, designed to achieve strong cryptographic properties. Additionally^7^, proposed an algorithm that leverages a novel chaotic system for S-box generation. Other approaches incorporate mathematical structures and optimization methods. Reference^8^ introduced coset graphs and symmetric groups as foundational elements for constructing robust S-boxes. Reference^9^ presented a chaos-based S-box technique for secure image encryption. Their improved coupling quadratic map efficiently eliminates fixed and reverse fixed points, thereby strengthening resistance to cryptographic attacks. Elliptic curves have also been widely adopted in S-box construction. Reference^10^ formulated an approach utilizing ordered elliptic curves over a prime field, achieving a time complexity of $\mathcal {O}(p^2)$ and a space complexity of $\mathcal {O}(p)$, where *p* represents the prime number associated with the elliptic curve. They later proposed an S-box generation method based on Mordell elliptic curves. Reference^11^ explored ordered isomorphic elliptic curves to develop secure S-boxes, requiring $\mathcal {O} (2^m)$ space and $\mathcal {O} (2^{m}p)$ time. Further refinement was provided by^12^, who leveraged elliptic curve properties such as ordering and isomorphism to enhance cryptographic strength. Reference^13^ applied Mordell elliptic curves in their S-box design methodology. Efficiency in large-scale S-box generation has also been a key research focus. Reference^14^ Ibrahim and Abbas (2021) introduced the fastest technique for producing an $m \times m$ S-box using large elliptic curves (ECs). Using given EC parameters and an integer *N*, the approach generates 3*N* points on the curve through two steps: first, a nondeterministic algorithm identifies *N* valid points on the curve; second, 2*N* more points are computed via the group law operation. These points are then utilized to perform swapping on an initial S-box, resulting in the final S-box. Experimental findings demonstrate that this method can quickly generate 8$\times$8 S-boxes across prime fields of varying bit sizes, with processing times reducing as bit length grows. However, the total computation time is largely influenced by the nondeterministic first step, which increases exponentially with *N*. A novel S-box generator is proposed by^15^, based on a deterministic algorithm over elliptic curves, to generate highly dynamic and secure S-boxes with low computational cost. Experimental results demonstrate that the generator is *i* times faster than the fastest available S-box generator^14^, where $i \in [6.720, 1.3 \times 10^5]$, for generating S-boxes of an input size $2^m, m = 4, 6, 8, 10, 12, 14, 16$ over large elliptic curves. This significant improvement enables significantly higher encryption throughput compared to the state-of-the-art schemes. Reference^16^ proposed a new pseudo random encryption key and constructed a novel S-boxes from the hyperchaotic 4D Chen system and the Mersenne Twister. An efficient and secure color image encryption scheme by^17^ based on a novel chaotic structure and dynamical strong S-box are built by full chaotic maps. Reference^18^ proposed a $16 \times 16$ S-box based on the hyperchaotic system with high nonlinearity and high cryptographic strength. A method proposed by^19^ generated an $8 \times 8$ S-box based on LA ring of order 1024 that is viewed as a large class of non associative rings. The use of a non associative LA ring enhances the robustness of the S-box. Reference^20^ proposes a new construction of substitution boxes $n \times n$ (S-boxes) using integer quaternion in order to enhance cryptographic strength. Reference^21^ designed a novel substitution box construction method using a chaotic fractional order nonlinear coupled map lattice system combined with an elementary cellular automaton. Reference^22^ introduced a method for designing dynamic S-boxes using an extended Two Dimensional Hyperchaotic Effect Coupled Map Lattice (2DHECML) system. The system is also used to enhance its chaotic behavior and cryptographic properties. Reference^23^ proposed two S-box methods based on the continuous time Lu Chen chaotic system for image encryption, which encompass row and column rotation and zigzag transformations.

Optimization techniques have played a crucial role in improving S-box cryptographic properties, particularly in maximizing nonlinearity. Several recent studies have focused on such methods to improve their resistance and overall performance. An efficient color image encryption algorithm was proposed by^24^ based on a two dimensional Sine Logistic Gaussian (2D-SLG) chaotic system and a multi-objective optimized S-box. The 2D-SLG system combines chaotic complexity with computational efficiency, while particle swarm optimization enhances the substitution performance with a nonlinearity of 106. This combination strengthens security without compromising encryption speed. Reference^25^ proposed a discrete chaotic map integrated with an improved Grey Wolf Optimizer (GWO), enhanced by a novel crossover operator to improve the optimization process. Their method produced high performance S boxes with a nonlinearity value of 106, highlighting the potential of combining chaotic systems with metaheuristic optimization for cryptographic purposes. An improved cryptographic system was developed by^26^ first employing ergodic chaotic maps to enhance the Particle Swarm Optimization (PSO) algorithm. The optimized PSO was then used to generate highly effective chaotic S-boxes, achieving an excellent nonlinearity value of 104. This approach demonstrates how chaotic systems can significantly boost the performance of both optimization algorithms and cryptographic components. Reference^27^ presented a new hybrid chaotic function created by connecting various one dimensional maps. The map’s performance was confirmed through Lyapunov exponent and entropy analysis. Additionally, the chaotic Jaya optimization algorithm was employed to create an optimized substitution box with a nonlinearity of 104. A new method combining a discrete chaotic map (DCM) with the firefly optimization algorithm (FOA) was introduced by^28^ to create cryptographically strong S-boxes. An initial S-box was generated using the DCM and then enhanced its nonlinearity through FOA. Their S-box achieved a nonlinearity value of 106, outperforming many other optimization based generators, though it remains below the optimal level. A method for designing robust S-boxes was introduced by^29^ in which chaos theory was combined with the teaching learning based optimization algorithm. They claimed that their resultant S-boxes have superior cryptographic properties than known techniques. However, the most significant drawback of this technique is that the cryptographic properties of their resultant S-box was achieved the nonlinearity value of 104 which are not near to ideal values. Reference^30^ proposed a metaheuristic approach that combines Ant Colony Optimization (ACO) with chaotic maps to construct a robust $8 \times 8$ substitution box (S-box). In this method, the initial S-box is transformed into a Traveling Salesman Problem (TSP) using an edge matrix. An optimal path, identified through ACO, directs the rotation of the intermediate S-box, resulting in a high-performance design with a nonlinearity value of 106. Reference^31^ introduced a 1D discrete chaotic map that leverages sensitivity to initial conditions and randomness in generated sequences to construct a strong S-box with a nonlinearity of 106. First, the chaotic map is iterated to generate initial S-box candidates, which then seed the population of a Cuckoo Search (CS) algorithm to further optimize cryptographic strength. Reference^32^ proposed a novel hybrid method using the Harris Hawks optimization algorithm (HHO) and the Booster algorithm for generating highly nonlinear S-boxes with low computational time. The (HHO) algorithm is employed in order to search in the large permutation search space to find an S-box of suitable cryptographic properties. Reference^33^ presented a robust S-box design through the use of the intrinsic physical randomness of phase noise and the SHA-256 hash function. The design method significantly improves security for the S-boxes along with improved performance in nonlinearity, SAC, and BIC tests. Reference^34^ designed a new technique for the construction of $8 \times 8$ S boxes that have selected cryptographic properties according to the ABO (African Buffalo Optimization) algorithm and a random function. Hybrid Adaptive Genetic Algorithm (HAGA)^35^, integrates genetic algorithms with local search to construct nonlinear S-boxes possessing strong cryptographic properties. The Chaotic Opposition based Learning Initialized Hybrid Algebraic-Heuristic (COBLAH) algorithm^36^, construct S-boxes by unifying algebraic methods with heuristic techniques using Galois field inversion, affine mapping, and genetic algorithms.

### Contributions

This paper aims to present a dynamic methodology for constructing S-boxes with superior cryptographic strength. The key contributions of this work are outlined as follows:

- A novel S-box generation technique is introduced, capable of rapidly producing highly dynamic and secure S-boxes. By leveraging binary sequences obtained from points on cubic Pell curves, this approach ensures efficient encryption with minimal computational overhead.
- A comprehensive security evaluation and comparative analysis confirm the superiority of the proposed S-box generator over existing techniques^10,11,14,15,37–49^.
- The proposed generator produces optimized S-boxes with higher nonlinearity than those generated by previous optimization methods^24–31^.
- With an optimal nonlinearity score of 108, the generated S-boxes provide both high-speed encryption and strong resistance against cryptographic attacks.
- The use of these optimized S-boxes significantly strengthens the security of image encryption, enhancing protection against statistical and differential attacks.

The paper is structured as follows: Section 2 covers fundamental concepts related to cubic Pell curves. Section 3 details the design and construction of the proposed S-box generators. Section 4 presents an in-depth security analysis and comparison with conventional S-box generators. Section 5 explores the practical implementation of the proposed generator in image encryption. Finally, Section 6 concludes the study with key findings and future research directions.

## Preliminaries

### Definition 1

Reference^3^: An integer $\alpha$ is considered a cubic residue modulo *p*, if there exists at least one solution to the congruence equation:

$$\begin{aligned} \mathscr {x}^3 \equiv \alpha \pmod {p} \end{aligned}$$

where a prime number *p* . If no such solution exists, $\alpha$ is a cubic non-residue modulo *p*.

### Definition 2

Reference^3^: An integer $\alpha$ is defined as a quadratic residue modulo $p^r$, if there exists at least one solution to the equation

$$\begin{aligned} \mathscr {x}^2 \equiv \alpha \pmod {p^r} \end{aligned}$$

where a prime number *p* and a non-negative integer *r*. If no such solution exists, $\alpha$ is a quadratic non-residue modulo $p^r$.

### Definition 3

Reference^3^: Cubic Pell Discrete Logarithm Problem (CPDLP):

Given a prime number *p*, a non-cubic integer $\alpha$ modulo *p*, a point $P_1(\mathscr {x}_1, \mathscr {y}_1, \mathscr {z}_1)$, and a point $P_{\lambda }(\mathscr {x}_{\lambda }, \mathscr {y}_{\lambda }, \mathscr {z}_{\lambda })$ on the curve with the cubic pell equation $\mathscr {x}^3 + \alpha \mathscr {y}^3 + \alpha ^2\mathscr {z}^3 - 3\alpha \mathscr {x}\mathscr {y}\mathscr {z} \equiv 1 \pmod {p}$, find $\lambda$, if any, such that $P_{\lambda }=\lambda P_1$.

The Cubic Pell Discrete Logarithm Problem (CPDLP) is as challenging as the Elliptic Curve Discrete Logarithm Problem (ECDLP).

### The theory of cubic Pell curves over fields

Let $\mathcal {K}$ be a field and $\alpha \in \mathcal {K}$. Consider the quotient ring $\mathcal {R}_{\alpha }= \frac{\mathcal {K}[t]}{(t^3 - \alpha )}$. It is known that $\mathcal {R}_{\alpha }$ forms a field if $\alpha$ is a cubic non-residue in $\mathcal {K}$.The expression for any element $\omega \in \mathcal {R}_{\alpha }$ can be written as $\omega = \mathscr {x} + \mathscr {y}\mathscr {t} + \mathscr {z}\mathscr {t}^2$, where $(\mathscr {x},\mathscr {y},\mathscr {z}) \in \mathcal {K}^3$. For two elements $\omega _1 = \mathscr {x}_1 + \mathscr {y}_1 \mathscr {t} + \mathscr {z}_1 \mathscr {t}^2$ and $\omega _{1} = \mathscr {x}_2 + \mathscr {y}_2 \mathscr {t} + \mathscr {z}_2 \mathscr {t}^2$ in $\mathcal {R}_{\alpha }$, their product is given by:

$$\omega _{1} \cdot \omega _{2} = \left[ \mathscr {x}_1 \mathscr {x}_2 + \alpha (\mathscr {y}_2 \mathscr {z}_1 + \mathscr {y}_1 \mathscr {z}_2)\right] + \left[ \mathscr {x}_2 \mathscr {y}_1 + \mathscr {x}_1 \mathscr {y}_2 + \alpha \mathscr {z}_1 \mathscr {z}_2\right] t + \left[ \mathscr {y}_1 \mathscr {y}_2 + \mathscr {x}_2 \mathscr {z}_1 + \mathscr {x}_1 \mathscr {z}_2\right] t^2 .$$

The norm of an element $\omega = \mathscr {x} + \mathscr {y}\mathscr {t} + \mathscr {z}\mathscr {t}^2$ is defined as^2^:

$$\begin{aligned} \mathcal {N}_\alpha (\omega ) = \mathscr {x}^3 + \alpha \mathscr {y}^3 + \alpha ^2 \mathscr {z}^3 - 3 \alpha \mathscr {x} \mathscr {y} \mathscr {z}. \end{aligned}$$

Now, define the set of unitary elements in $\mathcal {R}_{\alpha }$ as:

$$\mathfrak {U}_\alpha = \{\mathscr {x} + \mathscr {y}t + \mathscr {z}t^2 \in \mathcal {R}_{\alpha } \mid \mathscr {x}^3 + \alpha \mathscr {y}^3 + \alpha ^2 \mathscr {z}^3 - 3 \mathscr {x}\mathscr {y}\mathscr {z} = 1 \}.$$

This corresponds to the cubic Pell curve over $\mathcal {K}$, given by:

$$\begin{aligned} \mathcal{C}\mathcal{P}^3_\alpha (p): = \{ (\mathscr {x},\mathscr {y},\mathscr {z}) \in \mathcal {K}^3 \mid \mathscr {x}^3 + \alpha \mathscr {y}^3 + \alpha ^2 \mathscr {z}^3 - 3 \mathscr {x}\mathscr {y}\mathscr {z} = 1 \}. \end{aligned}$$

The natural product on $\mathfrak {U}_\alpha$ induces a generalized Brahmagupta product, which is defined for two points $\omega _1 = (\mathscr {x}_1, \mathscr {y}_1, \mathscr {z}_1)$ and $\omega _2 = (\mathscr {x}_2,\mathscr {y}_2, \mathscr {z}_2)$ in $\mathcal{C}\mathcal{P}^3_\alpha (p)$ as:

$$\omega _1 \oplus \omega _2 = \mathscr {x}_1 \mathscr {x}_2 + \alpha (\mathscr {y}_2 \mathscr {z}_1 + \mathscr {y}_1 \mathscr {z}_2) ,\mathscr {x}_2 \mathscr {y}_1 + \mathscr {x}_2 \mathscr {y}_1 + \alpha \mathscr {z}_1 \mathscr {z}_2 , \mathscr {y}_1 \mathscr {y}_2 + \mathscr {x}_2 \mathscr {z}_1 + \mathscr {x}_1 \mathscr {z}_2.$$

The set $(\mathcal{C}\mathcal{P}^3_\alpha (p), \oplus )$ forms a group with the identity element (1, 0, 0) . The inverse of any element $\omega = (\mathscr {x}, \mathscr {y}, \mathscr {z})$ in this group is given by:

$$\omega ^{-1} = (\mathscr {x}^2 - \alpha \mathscr {y}\mathscr {z}, \alpha \mathscr {z}^2 - \mathscr {x}\mathscr {y},\mathscr {y}^2 - \mathscr {x}\mathscr {z}).$$

#### Proposition 1

Reference^3^: Let $p^r$ represent a prime power and $\alpha \in \mathbb {Z}/ p^r \mathbb {Z}$. The set of solutions to the cubic Pell equation

$$\begin{aligned} \mathscr {x}^3 + \alpha \mathscr {y}^3 + \alpha ^2 \mathscr {z}^3 - 3 \alpha \mathscr {x} \mathscr {y} \mathscr {z} \equiv 1 \pmod {p^r}. \end{aligned}$$

is denoted by $\mathcal{C}\mathcal{P}_\alpha (p^r)$.

#### Lemma 1

Reference^3^: Let $p^r$ represent a prime power such that $p \equiv 1 \pmod 3$, with $r\ge 1$. Consider $\alpha \in \mathbb {Z}/ p^r \mathbb {Z}$ where gcd$(\alpha , p)=1$. The size of the set $\mathcal{C}\mathcal{P}_\alpha (p^r)$ is given by:

$$\left| \mathcal{C}\mathcal{P}_\alpha (p^r) \right| = {\left\{ \begin{array}{ll} (p-1)^2 p^{2(r-1)} & \\ \text {if } \alpha  \text { is a cubic residue modulo} ~p,\\ (p^2+p+1) p^{2(r-1)} & \\ \text {if }  \alpha~    \text {is a cube non-residue modulo}~p. \end{array}\right. }$$

## The proposed S-box generator based on cubic Pell curve

This section provides a detailed description of the dynamic S-box generator we have developed, highlighting the optimization techniques used to improve its performance. Our approach employs points derived from a cubic Pell curve to determine the locations for swapping operations within the initial S-box. By utilizing a deterministic method to generate these points, we effectively reduce the computational burden, improving efficiency compared to the approach proposed by^15^. This offers an alternative to the optimized S-box generators outlined in Table 10. The method presented here focuses on generating S-boxes with superior nonlinearity.

To explain the process, let $\mathcal{C}\mathcal{P}_\alpha (p^r)$ denote the set of solutions to the cubic Pell curve equation:

$$\begin{aligned} \mathscr {x}^3 + \alpha \mathscr {y}^3 + \alpha ^2 \mathscr {z}^3 - 3 \alpha \mathscr {x} \mathscr {y} \mathscr {z} \equiv 1 \pmod {p^r} \end{aligned}$$

where *p* is a prime number, $\alpha \in \mathbb {Z}/ p^r \mathbb {Z} \backslash \{0\}$ is a non-cubic integer, and *r* is a positive integer. Our generator starts with the non-identity point $G_i = (\mathscr {x}_i, \mathscr {y}_i, \mathscr {z}_i)$ $\in \mathcal{C}\mathcal{P}_\alpha (p^r)$ where *i* is the $i^{th}$ generated point. We generate *n* points $2G,3G,\ldots , nG$ over $\mathcal{C}\mathcal{P}_\alpha (p^r)$ by using the addition law^3^. For the two points $(\mathscr {x}_1, \mathscr {y}_1,\mathscr {z}_1)$ and $(\mathscr {x}_2, \mathscr {y}_2, \mathscr {z}_2) \in \mathcal{C}\mathcal{P}_\alpha (p^r)$. The addition law in $\mathcal{C}\mathcal{P}_\alpha (p^r)$ is defined as

1 $$\begin{aligned} (\mathscr {x}_3,\mathscr {y}_3, \mathscr {z}_3):= (\mathscr {x}_1,\mathscr {y}_1, \mathscr {z}_1) \oplus (\mathscr {x}_2,\mathscr {y}_2, \mathscr {z}_2) \end{aligned}$$

where

$$\begin{aligned} {\mathscr {x}_3} \equiv \alpha \mathscr {y}_1 \mathscr {z}_2 +\mathscr {x}_1 \mathscr {x}_2 + \alpha \mathscr {y}_2\mathscr {z}_1 \pmod {p^r}, \end{aligned}$$

$$\begin{aligned} {\mathscr {y}_3} \equiv \mathscr {x}_1 \mathscr {y}_2 + \alpha \mathscr {z}_1 \mathscr {z}_2 + \mathscr {x}_2 \mathscr {y}_1 \pmod {p^r}, \end{aligned}$$

$$\begin{aligned} {\mathscr {z}_3} \equiv \mathscr {x}_2 \mathscr {z}_1 + \mathscr {y}_1 \mathscr {y}_2 + \mathscr {x}_1 \mathscr {z}_2 \pmod {p^r}. \end{aligned}$$

For the generated *n* points $2G,3G,\ldots , nG$ on the cubic Pell curve, then calculate a new point $G'_i = (\mathscr {x}'_i, \mathscr {y}'_i, \mathscr {z}'_i)$ on each coordinate point of $G_i = (\mathscr {x}_i, \mathscr {y}_i, \mathscr {z}_i)$ as:

$$\begin{aligned} {\mathscr {x}'}:= \mathscr {y}_i \oplus \mathscr {z}_i , \end{aligned}$$

$$\begin{aligned} {\mathscr {y}'}:= \mathscr {x}_i \oplus \mathscr {z}_i , \end{aligned}$$

$$\begin{aligned} {\mathscr {z}'}:= \mathscr {x}_i \oplus \mathscr {y}_i . \end{aligned}$$

where $\oplus$ denote the XoR operation, and $\mathscr {x}_i, \mathscr {y}_i$ and $\mathscr {z}_i$ are the $i^{th}$ generated points using the addition law in the $\mathcal{C}\mathcal{P}_\alpha (p^r)$ Algorithm 1. Next, compute the binary representations $\textrm{bin}(\mathscr {x}'_{iG'}) = \mathscr {x}'_{i1}, \mathscr {x}'_{i2}, \dots , \mathscr {x}'_{it}$, $\textrm{bin}(\mathscr {y}'_{iG'}) = \mathscr {y}'_{i1}, \mathscr {y}'_{i2}, \dots ,\mathscr {y}'_{it'}$ and $\textrm{bin}(\mathscr {z}'_{iG'}) = \mathscr {z}'_{i1}, \mathscr {z}'_{i2}, \dots , \mathscr {z}'_{it''}$ of the ${\mathscr {x}'}, {\mathscr {y}'}$ and ${\mathscr {z}'}$ coordinates of the points $iG' = (\mathscr {x}'_{iG'}, \mathscr {y}'_{iG'},$ $\mathscr {z}'_{iG'})$, $i = 1, 2, \ldots , n$. Let $\textrm{bin}(iG') = \mathscr {x}'_{i1}, \mathscr {y}'_{i1}, \mathscr {z}'_{i1}, \dots , \mathscr {x}'_{ik},$ $\mathscr {y}'_{ik},\mathscr {z}'_{ik}$ denote the complete binary sequence $\mathcal {M}_i$ obtained by merging $\textrm{bin}(\mathscr {x}'_{iG'}), \textrm{bin}(\mathscr {y}'_{iG'})$, and $\textrm{bin}(\mathscr {z}'_{iG'})$, where $k = \min \{t, t', t''\}$. For the generated *n* points the resulting binary sequence $\mathcal {M}_i$ has a length of at least $m 2^m$, and divide this binary sequence $\mathcal {M}_i$ into $2^m$ subblocks, each of size *m* then onvert each *m*-bit subblock of $\mathcal {M}_i$ into its integer value, producing an integer sequence $\mathcal {I}_i$. Denote the current S-box *S* using the decimal values of the integer sequence $\mathcal {I}_i$. If this S-box *S* has repeated integer values, then *S* is not a bijective S-box. Our goal is to place all integer values in all positions of length $2^m$ without repetitions, ensuring we get a bijective S-box. Find the repeated integer values in the current S-box *S*. Set a sequence $S'$ and assign $-1$ to the repeated integer values of *S*. Generate a new sequence $T_s$ by assigning the number 1 to the corresponding position of $T_s$ if it appears in the index value of $S'$; otherwise, assign 0 to that position i.e $T_s[i]=1$ if $S'[i]=-1$, else $T_s[i]=0$. Generate a sequence *K* by assigning the missing index values of $T_s$. Set the final required S-box $S_b$ as initial S-box $S'$, where $\beta$ is the length of the sequence $S'$ and $\omega$ is the length of the sequence *K*. If the $S'[i]=-1$, then use the equation $\delta = 1+\mod ((\omega -j), S_{b}[i-1])$ to get the values. The dynamic S-box generated by our generator is represented by *S*, and its generation method is detailed in Algorithm 2.

The optimised S-box generator is designed by eliminating swap operations that minimize nonlinearity. The optimized S-box $S_{opt}$ is obtained by performing swapping operations that ensure high nonlinearity, as given in Algorithm 3.

**Algorithm 1:**
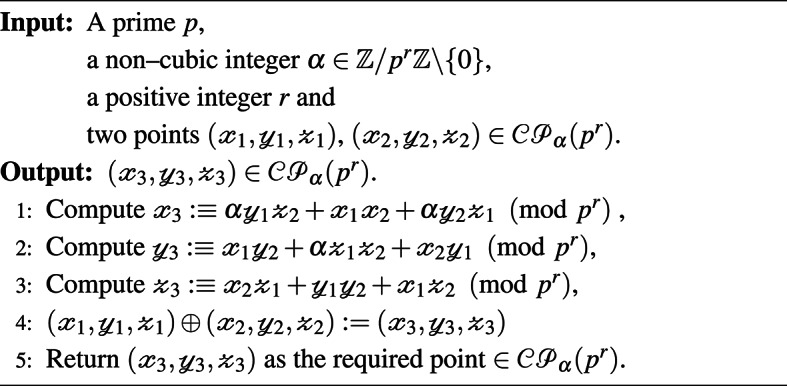
Addition in $$\mathcal{C}\mathcal{P}_\alpha (p^r)$$.

**Algorithm 2:**
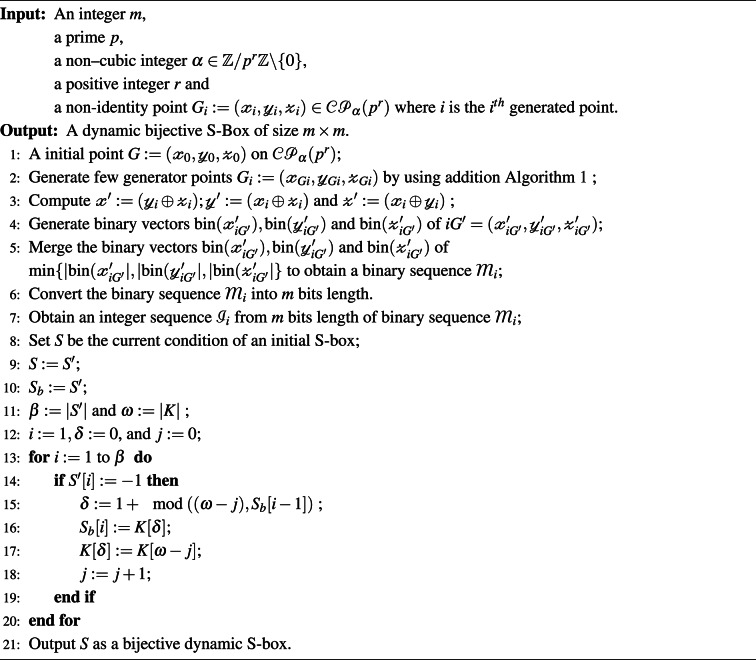
Dynamic S-Box Generation.

**Algorithm 3:**
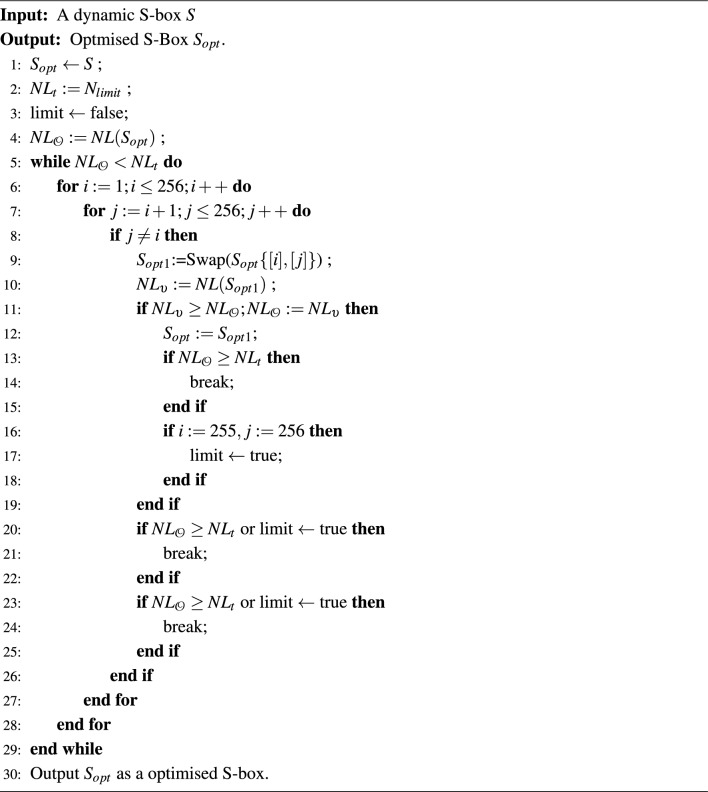
Optimised S-Box Generation.

## Security analysis and comparison

A comprehensive evaluation of our method was carried out, focusing on computational efficiency, dynamic behavior, and cryptographic strength. We compared the execution time of our S-box generation approach with the fastest known methods, specifically the one introduced by^15^. The cryptographic and dynamic characteristics of the S-boxes generated by our method were thoroughly examined and compared to those produced by other established techniques. In addition, we assessed the nonlinearity of the S-boxes and evaluated their security performance in relation to existing generators.

*Experimental setup*: The dynamic and optimized generated S-boxes used the recommended elliptic curve domain parameters from^50^, using primes of sizes 128, 224, 256, and 521 bits, generated with MATHEMATICA 14.0 on a machine with 16GB of RAM and an AMD Ryzen 7 5700U @ 1.80 GHz. We employed primes of different sizes with the parameters *p*, *c*, and *G* The parameters *p*, *c*, and *G* are detailed in the supplementary material.

### Experimental results and comparative analysis

####  Analysis of number of points

**Fig. 1:**
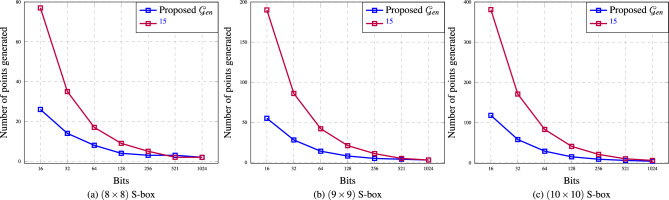
The number of points generated by the proposed S-box generator for $(8 \times 8)$, $(9 \times 9)$, and $(10 \times 10)$ S-boxes is compared with the generator by^15^.

Our experimental analysis focused on the number of points required to generate S-boxes of sizes $(8 \times 8)$, $(9 \times 9)$ and $(10 \times 10)$ using prime numbers with bit sizes of 16, 32, 64, 128, 256, 521, and 1024. The results indicate that our method generates [2, 26], [3, 55], and [4, 118] points for constructing $(8 \times 8)$, $(9 \times 9)$ and $(10 \times 10)$ S-boxes, respectively. In contrast, the method proposed by^15^ requires [2, 77], [3, 190], and [6, 381] points to generate the same S-box sizes. A comparison of the points generated by both methods is shown in Fig. 1, illustrating that our generator requires significantly fewer points than the method by^15^.

####  Computational time analysis

We measured the time taken to generate $(8 \times 8)$-S-boxes and $(10 \times 10)$-S-boxes using prime numbers with bit sizes of 128, 224, 256, and 521. The generation process was repeated five times, and the results showed that our generator produces an $(8 \times 8)$-S-box in the range of [0.0059, 0.0085] seconds and a $(10 \times 10)$-S-box in [0.0199, 0.0356] seconds. In comparison, the method by^15^ takes [0.00689, 0.00922] seconds and [0.0377, 0.06441] seconds, respectively. These results confirm that our proposed generator is significantly faster and more efficient than the current fastest method available.

The time complexity of the proposed dynamic S-box generator can be determined by evaluating each step separately of Algorithm 2. Line 1: Taking a non-identity point $G:= (\mathscr {x}_0, \mathscr {y}_0, \mathscr {z}_0)$ on the cubic Pell curve the time is $\mathcal {O}(1)$. Line 2: The generation of *n* points on the cubic Pell curve using the addition law with Algorithm 1 repeatedly, has a time complexity of $\mathcal {O}(n \cdot \log p)$, as each addition requires a fixed number of modular arithmetic operations. Line 3: Compute the modified coordinates $(\mathscr {x}'_i, \mathscr {y}'_i, \mathscr {z}'_i)$ using XOR operations has a time complexity of $\mathcal {O}(n)$. Line 4: Conversion of $(\mathscr {x}'_i, \mathscr {y}'_i, \mathscr {z}'_i)$ into binary sequences, which takes $\mathcal {O}(n \cdot \log p )$ time as each coordinate is expressed with $\log p$ bits. Lines 5 to 8: The process of merging and partitioning the binary sequences into $2^m$ sub-blocks, along with converting them into decimal values to form the initial S-box requires $\mathcal {O}(2^m)$ time. Lines 9 to 20: Detecting and replacing duplicate entries to ensure bijectivity requires a linear traversal of $2^m$ entries, adding an additional $\mathcal {O}(2^m)$ time. Since $2^m$ is a small fixed constant (256 for an $8 \times 8$ S-box) and $n \ll p$ (i.e that the number of points *n* generated on the cubic Pell curve is significantly smaller than the underlying prime *p*). Therefore, the overall time complexity of the proposed dynamic S-box generator is

$$\mathcal {O}(n \cdot \log p+2^m) \approx \mathcal {O}(\log p)$$,

for fixed $m=8$ and the number of points *n* is also small in the range [2, 26].

### Dyanmic S-box performance analysis

We generated 10,000 dynamic S-boxes using the approach outlined in Section 3 and conducted an extensive security evaluation to assess their cryptographic strength. This evaluation included a variety of important properties, such as nonlinearity (NL), differential approximation probability (DAP), strict avalanche criterion (SAC), linear approximation probability (LAP), bit independence criterion (BIC), and algebraic complexity (AC).

#### Nonlinearity (NL)

NL of an S-box is a crucial factor in enhancing the confusion between the ciphertext and plaintext, improving its cryptographic security. One of the primary measures of the effectiveness of an S-box is its nonlinearity, as defined by^51^. NL is quantified as the minimum Hamming distance between the S-box function $\sigma :Z_2{^m} \rightarrow Z_2{^m}$ and the set of all affine functions. This property plays a pivotal role in the security of the encryption system, as it helps prevent attackers from exploiting linear relationships between inputs and outputs. Mathematically, the nonlinearity of an S-box is given by:

2 $$\begin{aligned} NL(S) \triangleq \min _{(\omega , \alpha , \beta )} \{ {x} \in Z_2^{m} \vert \omega .S(x) \ne (\beta \oplus \alpha .x)\}, \end{aligned}$$

where $\omega \in Z_2^{m},\alpha \in Z_2^{m}$ and $\beta \in Z_2$. The NL(*S*) is required to be as close as possible to its maximum value, given by $(2^{m-1}-2^{\frac{m}{2}-1})$ for perfectly non-linear functions^51^.

The nonlinearity of an S-box can be determined by computing its Walsh spectrum. The Walsh spectrum is defined as:

3 $$\begin{aligned} \mathcal{W}\mathcal{S}(\omega ) \triangleq \sum _{\alpha \in Z_2^{m}} (-1)^{S(x) \oplus \alpha .\omega }, \end{aligned}$$

where $\omega \in Z_2^{m}$, and $\alpha .\omega$ denotes the dot product, calculated as $\alpha .\omega = \alpha _1\oplus \omega _1,........ \alpha _n\oplus \omega _n$. Based on this, the nonlinearity *NL*(*S*) of the S-box is given by:

4 $$\begin{aligned} NL(S) \triangleq 2^{m-1} (1-2^{-m} \max _{\alpha \in Z_2^{m}}\vert \mathcal{W}\mathcal{S}(\omega ) \vert ) \end{aligned}$$

**Fig. 2:**
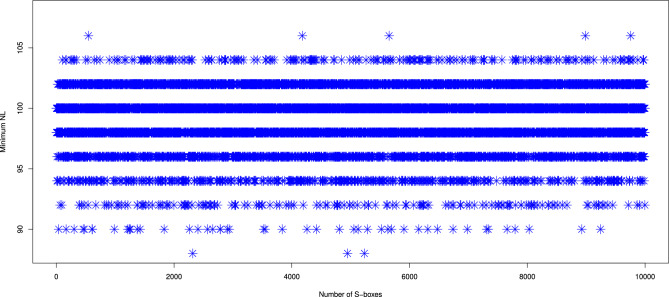
Results of nonlinearity with eight boolean functions for 10,000 randomly generated S-boxes.

The nonlinearity values with respect to eight boolean functions of the 10,000 S-boxes generated by our method are shown in Fig. 2. As reflected in Table 1, 93% of the generated S-boxes exhibited a nonlinearity at least 96. This demonstrates that our proposed generator is highly efficient in producing S-boxes that offer strong resistance to linear cryptanalysis, reinforcing their cryptographic security. We compared the nonlinearity due to eight boolean functions of 10,000 S-boxes produced by our method with those produced by several existing generators, including those from^5,10,14,15,42,44,52^ as shown in Table 2. The results revealed that our generator consistently outperformed the others in terms of minimum nonlinearity across all generated S-boxes. Moreover, the maximum nonlinearity achieved by our method was also higher than that of other approaches, including those by^5,10,42,44^, further demonstrating its superior capacity for generating cryptographically secure S-boxes.

The S-boxes ($\mathcal {S}_{\mathcal {P}})$, listed in Table 3, reached the optimal nonlinearity due to eight boolean functions of 106 with the input parameters $(m, p, \alpha ,r,G_i)$, where,

$$m=8, \alpha =9, r=2$$,

$$p= 11579208923731619542357098500868790785326998466564056403945758400 7908834671663$$,

$$x_{G_1} = {78678897980110680189937615394517648981960744210695720077288090136718124162276}$$,

$$y_{G_1} = {105316748987465574731669528188867710098205080952350007334827872052771392502134},$$

$$z_{G_1} = {15955009163358243145477188650123601990859004833262966782397166421349909202929}$$.

Additionally, we compared the nonlinearity due to eight boolean functions and 256 boolean functions of our dynamic S-box $(\mathcal {S}_{\mathcal {P}})$ to that of other methods listed in Table 4. This solidifies the proposed generator’s effectiveness in cryptographic applications, outperforming methods presented by^16–18,20–22,37,39,44,47–49^.

**Table 1:** The percentage of nonlinearity with eight boolean functions achieved by the proposed generator for 10,000 S-boxes.

NL	88	90	92	94	96	98	100	102	104	106
No. of S-boxes	3	48	160	468	1140	2472	3375	2099	230	5

**Table 2:** Comparison of the nonlinearity over eight boolean functions results of 10,000 randomly generated S-boxes.

S-boxes	Nonlinearity over eight boolean functions	
	Min.	Max.
Proposed Method	88	106
Method^15^	88	106
Method^52^	80	106
Method^14^	84	106
Method^42^	84	104
Method^10^	64	102
Method^5^	82	104
Method^44^	82	104

**Table 3:** The dynamic S-box $(\mathcal {S}_{\mathcal {P}})$ by proposed method.

235	15	69	218	114	178	167	31	84	205	88	118	39	138	175	241
79	13	119	48	219	236	29	145	234	116	53	117	136	20	62	2
7	67	246	110	41	221	140	36	11	37	44	199	30	133	105	38
22	222	122	98	135	146	147	12	173	89	197	213	107	153	8	148
25	139	26	74	49	132	57	155	206	81	170	80	150	73	123	93
21	247	195	77	204	181	228	166	211	112	193	127	75	191	33	216
238	227	101	254	19	202	159	224	164	109	233	163	16	17	35	232
63	169	174	58	161	245	223	192	50	141	208	168	156	42	120	237
76	226	55	100	86	10	34	97	85	201	248	162	91	71	190	198
243	217	130	66	188	28	210	134	54	14	72	154	96	252	87	83
23	64	70	111	99	3	209	137	239	152	126	186	158	4	18	220
51	5	214	60	46	143	129	244	179	242	40	106	128	125	231	183
144	184	180	151	121	78	102	142	149	203	94	240	92	56	157	113
215	24	65	187	251	104	124	171	59	249	131	43	9	172	90	6
115	82	200	108	1	185	45	103	182	230	207	68	95	229	255	253
165	225	0	189	27	176	61	52	196	47	250	194	160	177	212	32

**Table 4:** Comparison between the proposed dynamic S-box ($\mathcal {S}_{\mathcal {P}})$ and other existing S-box generators.

S-boxes	Nonlinearity over eight boolean functions	Nonlinearity over 256 boolean functions	LAP	DAP	AC	SAC		BIC-SAC		BU	AI
						Min	Max	Min	Max		
Proposed S-box $(\mathcal {S}_{\mathcal {P}})$	106	94	0.133	0.047	255	0.375	0.594	0.484	0.525	20	104
^16^	100	86	0.164	0.039	255	0.421	0.578	0.471	0.527	20	104
^17^	98	94	0.133	0.039	251	0.391	0.578	0.476	0.519	18	96
^18^	102	92	0.140	0.047	254	0.391	0.594	0.457	0.533	22	88
^20^	104	92	0.141	0.047	255	0.406	0.593	0.460	0.533	22	104
^21^	102	94	0.133	0.047	254	0.375	0.609	0.478	0.519	20	88
^22^	104	94	0.133	0.039	255	0.421	0.625	0.466	0.533	18	96
^37^	104	84	0.172	0.047	254	0.422	0.578	0.428	0.531	24	104
^38^	106	92	0.141	0.039	254	0.391	0.625	0.469	0.529	20	104
^39^	104	94	0.133	0.047	254	0.352	0.586	0.477	0.521	20	96
^40^	106	94	0.133	0.047	254	0.406	0.594	0.469	0.529	18	96
^41^	106	94	0.133	0.039	255	0.406	0.609	0.482	0.541	18	96
^42^	106	94	0.148	0.039	254	0.422	0.594	0.471	0.533	20	104
^43^	106	90	0.148	0.047	254	0.391	0.609	0.472	0.525	18	96
^10^	106	94	0.148	0.047	255	0.437	0.593	0.464	0.544	20	100
^44^	104	92	0.141	0.054	253	0.406	0.594	0.461	0.522	44	120
^46^	106	90	0.148	0.047	255	0.406	0.641	0.471	0.537	20	112
^47^	104	94	0.145	0.039	255	0.391	0.625	0.471	0.531	20	104
^11^	106	80	0.188	0.039	253	0.406	0.609	0.465	0.527	24	96
^49^	96	90	0.148	0.047	254	0.391	0.625	0.477	0.531	22	104
^48^	100	94	0.152	0.039	255	0.391	0.586	0.468	0.537	22	96
^53^	106	94	0.152	0.039	255	0.391	0.586	0.468	0.537	18	104
^52^	106	90	0.152	0.047	255	0.391	0.586	0.468	0.537	24	96

#### Linear approximation probability (LAP)

LAP was first introduced by^54^, quantifies the probability that a specific input bit correlates with a corresponding output bit in a cryptographic function. This metric helps assess the strength of a cipher by evaluating the potential for linear relationships between inputs and outputs, which could lead to vulnerabilities if the probability is too high. To calculate the LAP of the S-box, we can apply the following formula:

5 $$\begin{aligned} LAP(S)\triangleq \frac{1}{2^{n}}\{\max _{\alpha ,\beta \ne 0} \left| N(\alpha ,\beta ) \right| \}, \end{aligned}$$

where $N(\alpha ,\beta ) \triangleq {\#\{ {x} \in Z_2^{n} \vert x \cdot \alpha =S(x) \cdot \beta \}}-2^{n-1}, \alpha \in Z_2^{n}$ and $\beta \in Z_2^{n}$. Smaller LAP(*S*) values provide higher resistance to linear cryptanalysis.

**Fig. 3:**
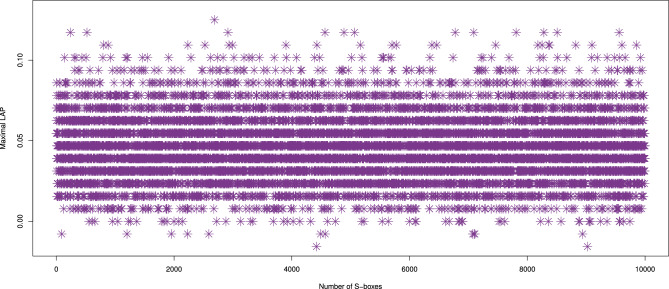
The LAP results of 10,000 randomly generated S-boxes.

LAP values for the 10,000 S-boxes produced by our approach are presented in Fig. 3, with a range from [-0.0156, 0.1406] and an average of 0.0466. These results suggest that our S-boxes exhibit a strong resistance to linear cryptanalysis. In comparison with other S-box generators, as shown in Table 4, the LAP values of our S-boxes are significantly lower. This indicates that our approach yields more secure S-boxes, with reduced susceptibility to linear attacks, compared to the methods in^10,11,16–18,20–22,37–44,46–49,52,53^.

#### Differential approximation probability (DAP)

DAP measure, introduced by^55^, evaluates the susceptibility of an S-box to differential cryptanalysis. An S-box with high differential uniformity is considered more secure against such attacks. A lower DAP value implies that the S-box has a greater ability to resist differential cryptanalysis, making it more robust in cryptographic applications. The DAP is calculated as follows:

6 $$\begin{aligned} DAP(S) \triangleq \max _{\delta {x} \ne 0,\delta {y}\in Z_2^{n} } \left( \frac{ D (\delta {x},\delta {y})}{2^{n}} \right) \end{aligned}$$

where $$D(\delta {x},\delta {y})\triangleq \#\{ {x} \in Z_2^{n} \vert S(x)\oplus S(x\oplus \delta {x}) =\delta {y}\}, \delta {x} \in Z_2^{n}$$ and $$\delta {y} \in Z_2^{n}$$.

**Fig. 4:**
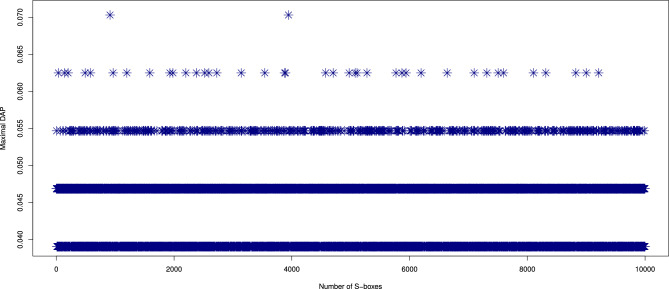
The DAP value results of 10,000 randomly generated S-boxes.

DAP values for the 10,000 S-boxes produced by our proposed generator are shown in Fig. 4. The DAP values for all the S-boxes lie within the ideal range of [0.039, 0.063], indicating strong resistance to differential cryptanalysis. The DAP of the proposed S-box ($\mathcal {S}_{\mathcal {P}}$) is 0.047. The DAP results for the proposed S-box are presented in Table 4, indicating that its performance is comparable to that of the existing S-box generators^10,11,16–18,20–22,37–44,46–49,52,53^.

####  Strict avalanche criterion (SAC)

SAC, introduced by^56^, is a method for testing S-boxes that combines the concepts of avalanche behavior and completeness. It measures the ability of the S-box to induce confusion in the cipher. An S-box satisfies the SAC if a small variation in the input bits leads to a change in the output bits with a probability of 0.5, ensuring that the cipher is highly sensitive to input alterations.

7 $$\begin{aligned} \mathcal {S}(i,j)\triangleq \frac{1}{2^{n}}\left[ \ (S_i(\nu )\oplus \omega (S_i(\nu \oplus \delta _j)))\right] , \end{aligned}$$

where $\omega (\delta _j)=1$ is the number of non-zero bits in $\delta _j$, $1\le i,j \le 8$ and $\delta _j \in Z_2^{n}$.

**Fig. 5:**
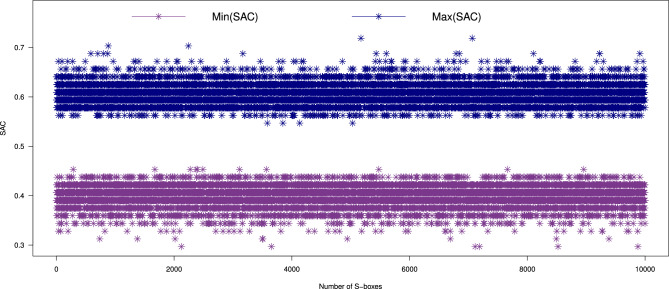
The maximum and minimum SAC values of 10,000 randomly generated S-boxes.

**Fig. 6:**
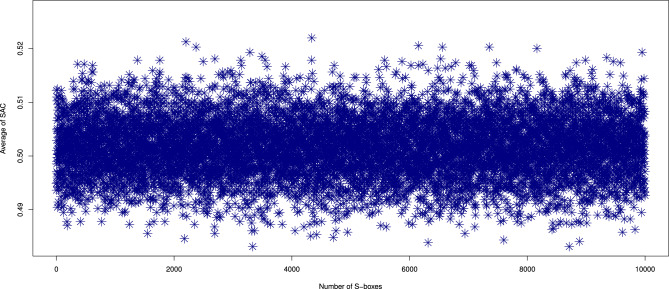
Average SAC results of 10,000 randomly generated S-boxes.

We conducted an extensive SAC analysis on 10,000 S-boxes generated by our proposed method. The results, shown in Figs. 5 and  6, highlight the average, minimum, and maximum SAC values. The SAC values for the generated S-boxes were found to fall within the ranges of [0.2969, 0.4531] for the minimum, [0.5469, 0.7188] for the maximum, and [0.3982, 0.6049] on average. These values are in close proximity to the ideal value of 0.5, confirming that the generated S-boxes comply with SAC requirements. This ensures that the S-boxes offer significant resistance to attacks exploiting Boolean functions. Furthermore, a comparison of the SAC values for our S-boxes with other generators is provided in Table 4, showing that our method performs favorably compared to existing methods^10,11,16–18,20–22,37–44,46–49,52,53^.

#### Algebraic complexity (AC)

**Fig. 7:**
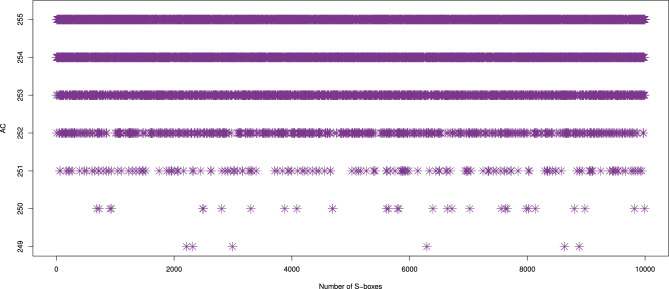
AC results of 10,000 randomly generated S-boxes.

The concept of AC was introduced by^57^ as a key measure for evaluating the resistance of an S-box to algebraic attacks. The AC of an S-box refers to the number of non-zero terms in its corresponding polynomial representation. The higher the AC, the more difficult it is for an attacker to perform algebraic attacks successfully. S-boxes with higher AC values provide a stronger cryptographic defense, with a maximum AC value of 255 indicating significant protection against such attacks. Thus, a higher AC value is crucial for enhancing the cryptographic security of S-boxes. The AC values of the 10,000 S-boxes, ranging from 249 to 255, are depicted in Fig. 7. From Table 5, it is clear that over 99% of our generated S-boxes have an AC value $\ge 251$, which is higher than^58^. Additionally, Table 4 compares the AC results of the S-box ($\mathcal {S}_{\mathcal {P}}$) with the other S-box generators^10,11,16–18,20–22,37–44,46–49,52,53^.

**Table 5:** The AC results of 10,000 S-boxes generated by the proposed generator are compared with the method presented in^58^.

AC	Number of S-boxes	
	Proposed	Reference^58^
247	0	3
248	0	4
249	6	10
250	30	18
251	157	172
252	630	655
253	1759	1762
254	3662	3635
255	3756	3741

#### Bit independence criterion (BIC)

BIC, introduced by^56^, is a method for evaluating how the output bits of an S-box change when a single input bit is modified. This criterion, similar to the strict avalanche criterion, is used to assess the resilience of an S-box against potential Boolean function-based attacks.

**Fig. 8:**
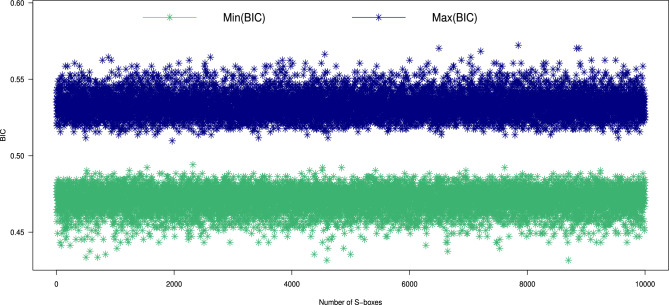
The maximum and minimum BIC values of 10,000 randomly generated S-boxes.

**Fig. 9:**
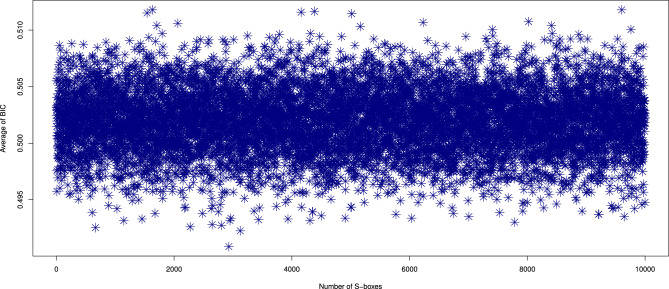
Average BIC results of 10,000 randomly generated S-boxes.

To analyze the BIC, we computed the values for 10,000 S-boxes generated by our proposed method. The results, including the minimum, maximum, and average BIC values, are illustrated in Figs. 8, 9. The computed ranges for the BIC values are: average [0.4708, 0.5332], minimum [0.4316, 0.4941], and maximum [0.5098, 0.5723]. Additionally, we compared the BIC values of the S-box generated by our method ($\mathcal {S}_{\mathcal {P}})$ with those of other S-box generators^10,11,16–18,20–22,37–44,46–49,52,53^, as listed in Table 4. The comparison indicates that our proposed generator excels at producing dynamic S-boxes with strong BIC values, reinforcing their effectiveness in cryptographic applications.

#### Absolute indicator (AI)

An absolute indicator of a boolean function *f* is defined as the largest absolute value of auto correlation is given by^59^

8 $$\begin{aligned} \mathcal {A}_\mathscr {f} \triangleq \max _{\gamma \ne 0,\gamma \in Z_2^{m} }\left| \Lambda _f(\gamma ) \right| , \end{aligned}$$

where $\Lambda _f(\gamma )$ is the autocorrealtion of the function *f* over $Z_2^{m}$ is calculated as

$$\begin{aligned} \Lambda _f(\gamma ) \triangleq \sum _{\mathscr {x} \in Z_2^{m}} (-1)^{f(x) \oplus f(x+\gamma )}. \end{aligned}$$

The comparison of the AI values for our proposed dynamic S-box $(\mathcal {S}_{\mathcal {P}})$ with other generators is provided in Table 4, showing that our method performs favorably compared to existing methods^44,46^.

#### Boomerang uniformity (BU)

The concept of boomerang uniformity was introduced by^60^, is defined as the maximum value in the boomerang connectivity table, excluding the row and column corresponding to index 0.

9 $$\begin{aligned} \mathcal {B_U} \triangleq \max _{\alpha , \beta \ne 0,\alpha , \beta \in Z_2^{m} }\left| \mathcal {B_S}(\alpha , \beta ) \right| , \end{aligned}$$

where $\mathcal {B}_\mathscr {c}(\alpha , \beta )$ is the boomerang connectivity table of $\mathcal {S}$ is given by

$$\begin{aligned} \mathcal {B}_\mathscr {c}(\alpha , \beta ) \triangleq \#\{ {x} \in Z_2^{m} \vert S^{-1}(S(x)\oplus \beta ) \oplus S^{-1}(S(x \oplus \alpha )\oplus \beta )=\alpha \}, \alpha , \beta \in Z_2^{m}. \end{aligned}$$

The comparison of the BU values for our proposed dynamic S-box $(\mathcal {S}_{\mathcal {P}})$ with other generators is provided in Table 4, showing that our method performs favorably compared to existing methods^11,18,20,37,44,48,49,52^.

### Optimized S-box performance analysis

In order to generate optimized S-boxes of size $(8 \times 8)$, we leveraged elliptic curve domain parameters as suggested by^50^, utilizing prime sizes of 128, 224, 256, and 521 bits. The optimized S-boxes were constructed by applying the swapping procedure outlined in Algorithm 3, resulting in a nonlinearity value of 108. The generated optimized S-boxes $\mathcal {S}_{128}, \mathcal {S}_{224}, \mathcal {S}_{256},$ and $\mathcal {S}_{521}$ are presented in Tables 6, 7, 8 and 9.

1. A comparison of the NL due to eight boolean functions and 256 boolean functions of our optimized S-boxes with those of existing optimized S-box generators^24–31,33,34^ is provided in Table 10. Our optimized S-boxes $\mathcal {S}_{128}$, $\mathcal {S}_{224}$, $\mathcal {S}_{256}$, and $\mathcal {S}_{521}$, with a nonlinearity due to eight boolean functions of 108, show superior performance in comparison with other generators^24–31,33,34^, confirming the high nonlinearity achieved by our method.
2. LAP values of the optimized S-boxes $\mathcal {S}_{128}, \mathcal {S}_{224}$, $\mathcal {S}_{256},$ and $\mathcal {S}_{521}$ are evaluated and listed in Table 10. These values are notably higher than those of existing S-box generators^24–31,33,34^, indicating that the optimized S-boxes produced by our method exhibit stronger resistance to linear cryptanalysis.
3. DAP values for the optimized S-boxes $\mathcal {S}_{128}, \mathcal {S}_{224}, \mathcal {S}_{256},$ and $\mathcal {S}_{521}$ are presented in Table 10. The DAP values for the optimized S-boxes $\mathcal {S}_{128}, \mathcal {S}_{224}, \mathcal {S}_{256},$ and $\mathcal {S}_{521}$ are 0.047, 0.039, 0.047, and 0.039. The DAP results for the proposed S-box are presented in Table 10, indicating that its performance is comparable to that of the existing S-box schemes^24–31,33,34^, making them more resilient against differential cryptanalysis attacks.
4. AC values of our optimized S-boxes $\mathcal {S}_{128}, \mathcal {S}_{224}, \mathcal {S}_{256},$ and $\mathcal {S}_{521}$, shown in Table 10, outperform those from other generators^24–31,33,34^. Higher AC indicates that the proposed S-boxes are more secure against algebraic attacks, providing enhanced cryptographic strength.
5. BU and AI values of our optimized S-boxes $\mathcal {S}_{128}, \mathcal {S}_{224}, \mathcal {S}_{256},$ and $\mathcal {S}_{521}$, shown in Table 10, perform favorably compared to existing methods^24–31,33,34^.
6. SAC values of our optimized S-boxes $\mathcal {S}_{128}, \mathcal {S}_{224}, \mathcal {S}_{256},$ and $\mathcal {S}_{521}$ are close to the ideal value of 0.5, demonstrating strong confusion properties. As indicated in Table 10, these S-boxes have better SAC results than compared to other S-box generation schemes^24–31,33,34^. Therefore, the S-boxes $\mathcal {S}_{128}, \mathcal {S}_{224}, \mathcal {S}_{256},$ and $\mathcal {S}_{521}$ demonstrate resistance to cryptanalysis by the Boolean function.
7. Finally, BIC values of the optimized S-boxes $\mathcal {S}_{128}, \mathcal {S}_{224}$, $\mathcal {S}_{256},$ and $\mathcal {S}_{521}$, as presented in Table 10, are found to be comparable to those from other state-of-the-art S-box generation schemes^24–31,33,34^. This confirms that our method produces optimized S-boxes with excellent BIC performance, ensuring robust cryptographic security.

**Table 6:** The generated S-box, $\mathcal {S}_{128}$.

64	4	109	203	110	184	7	69	39	171	180	166	128	66	19	0
42	10	219	105	49	250	87	96	205	47	188	8	137	93	197	71
236	86	201	224	123	211	145	139	52	143	106	241	173	116	189	101
20	221	228	37	246	27	130	95	38	248	5	154	132	104	3	44
142	111	135	98	164	32	70	103	192	74	179	183	182	83	175	194
34	233	240	253	77	254	48	243	160	125	84	12	206	54	222	6
22	62	156	249	165	99	159	174	231	223	35	214	82	45	122	191
115	50	112	133	46	26	76	187	97	232	55	144	88	11	36	13
41	245	43	152	239	68	114	1	148	244	255	186	217	170	67	167
73	193	91	215	15	24	65	162	199	216	251	134	17	51	80	181
140	157	237	138	127	25	72	195	85	78	61	176	79	207	113	18
229	141	121	153	16	155	196	204	242	60	63	247	185	30	90	163
94	31	2	190	168	161	14	198	29	9	75	100	177	252	23	220
33	210	119	235	150	21	158	149	117	146	126	118	89	53	202	107
136	200	209	124	57	238	58	147	102	120	28	56	169	40	208	81
131	129	230	234	108	92	178	213	225	227	172	218	151	59	226	212

**Table 7:** The generated S-box, $\mathcal {S}_{224}$.

193	69	116	181	245	82	225	62	231	137	204	150	81	132	227	247
129	110	99	47	244	197	35	80	221	94	234	229	139	212	119	56
34	102	134	104	59	222	83	224	213	6	195	29	75	184	120	16
206	97	78	233	237	127	158	46	143	10	173	85	14	100	26	0
3	228	147	60	172	251	19	190	45	9	41	135	182	84	235	223
157	72	128	105	68	160	236	20	58	131	15	49	249	61	1	254
38	63	164	23	124	42	232	112	218	192	211	144	39	118	138	242
36	108	101	174	187	89	215	246	126	238	230	53	255	148	183	4
219	130	21	64	48	189	163	93	25	186	165	121	113	151	77	28
74	98	214	185	24	22	202	90	117	73	52	155	141	115	142	252
123	159	5	88	2	51	154	7	168	54	170	79	95	18	179	156
220	152	226	216	70	166	162	241	153	175	96	205	146	12	31	217
133	239	106	250	145	66	55	103	67	253	92	107	177	33	208	207
167	86	32	171	199	91	169	50	8	176	180	37	140	240	13	44
209	200	194	40	17	203	109	201	87	243	161	149	198	30	27	11
248	114	191	71	188	210	178	43	125	122	57	111	65	76	136	196

**Table 8:** The generated S-box, $\mathcal {S}_{256}$.

228	169	10	222	217	132	147	170	135	88	215	253	50	68	232	185
95	47	93	84	111	196	17	241	59	153	49	128	251	194	221	213
129	246	231	250	4	179	247	252	189	18	34	61	190	206	79	157
77	82	156	202	238	80	19	36	99	57	130	6	159	120	104	83
211	152	174	127	109	14	223	144	7	26	188	58	13	175	195	87
160	204	124	248	67	173	101	60	154	150	226	187	42	40	73	103
96	136	53	164	27	75	216	214	112	37	142	186	168	108	236	46
91	70	178	52	64	11	192	167	63	163	166	201	2	81	45	143
234	183	229	107	76	0	172	126	97	24	65	225	71	72	105	220
44	56	212	16	31	227	233	242	254	5	237	3	35	98	165	90
85	200	149	48	134	240	89	155	205	235	207	9	21	115	1	140
33	116	244	122	69	218	106	191	23	199	100	125	171	133	245	117
203	41	230	184	148	54	32	114	28	210	141	38	197	193	177	30
86	113	219	110	39	138	255	139	92	119	239	62	66	94	8	78
25	43	181	243	198	146	12	51	29	15	121	158	176	162	182	180
208	131	22	209	145	74	20	161	249	123	55	151	102	224	137	118

**Table 9:** The generated S-box, $\mathcal {S}_{521}$.

75	179	171	17	116	185	44	87	202	13	19	102	16	121	150	99
25	221	118	113	224	145	58	177	21	71	92	48	83	209	107	80
37	181	180	55	227	243	223	1	3	114	104	14	245	254	167	53
131	140	186	213	219	89	103	59	20	136	188	78	160	36	122	60
61	183	143	196	130	142	197	134	249	236	40	50	155	52	35	162
70	255	244	192	119	193	10	225	169	110	228	29	94	56	123	237
170	235	106	54	95	90	112	149	191	147	18	46	175	251	204	220
159	146	68	64	7	241	49	23	208	72	141	98	125	250	201	207
253	133	65	73	76	194	187	66	12	229	81	240	41	168	91	120
214	158	161	6	31	174	242	45	5	39	26	85	234	198	124	109
100	247	9	43	138	42	135	151	132	57	2	38	105	173	126	139
51	218	154	199	239	190	97	153	84	11	127	164	246	165	67	203
156	184	15	238	30	82	163	88	216	28	22	215	96	217	33	108
212	24	166	211	101	157	230	232	117	233	79	77	195	0	47	252
86	206	74	222	8	32	63	182	34	69	152	226	137	129	172	200
231	62	111	205	178	144	4	128	115	189	176	148	93	210	27	248

**Table 10:** Comparison of the proposed optimized S-boxes $\mathcal {S}_{128}, \mathcal {S}_{224}$, $\mathcal {S}_{256},$ and $\mathcal {S}_{521}$ with existing generators.

S-boxes	Nonlinearity over eight boolean functions	Nonlinearity over 256 boolean functions	LAP	DAP	AC	SAC		BIC-SAC		BU	AI
						Min	Max	Min	Max		
S-box $\mathcal {S}_{128}$	108	96	0.125	0.047	254	0.359	0.578	0.482	0.531	24	96
S-box $\mathcal {S}_{224}$	108	96	0.125	0.039	254	0.422	0.609	0.475	0.527	18	96
S-box $\mathcal {S}_{256}$	108	96	0.125	0.047	255	0.422	0.578	0.471	0.529	20	96
S-box $\mathcal {S}_{521}$	108	96	0.125	0.039	255	0.406	0.593	0.482	0.527	20	96
^33^	104	92	0.141	0.047	254	0.406	0.578	0.466	0.535	20	112
^34^	106	94	0.132	0.047	253	0.406	0.609	0.471	0.531	18	96
^24^	106	92	0.141	0.039	254	0.421	0.593	0.459	0.529	18	96
^25^	106	94	0.133	0.039	255	0.390	0.594	0.475	0.527	24	112
^26^	104	92	0.141	0.039	254	0.406	0.641	0.469	0.527	20	112
^27^	104	94	0.133	0.039	255	0.359	0.609	0.457	0.535	18	104
^28^	106	94	0.133	0.039	251	0.422	0.578	0.465	0.525	24	96
^29^	104	94	0.133	0.039	254	0.438	0.641	0.475	0.547	18	96
^30^	106	90	0.148	0.039	255	0.406	0.562	0.471	0.523	20	96
^31^	106	94	0.133	0.039	255	0.406	0.578	0.459	0.531	20	96

###  NIST statistical randomness test

The statistical test results for randomness carried out using the tool provided by NIST 800-22^61^ on the roposed S-boxes $\mathcal {S}_{p}$, $\mathcal {S}_{128}$, $\mathcal {S}_{224}$, $\mathcal {S}_{256}$ and $\mathcal {S}_{521}$. These tests are designed to find any non-randomness in the range [0,255] of the output sequence. Each S-box contains 256 values ranging from 0 to 255 were transformed into binary sequences to form the input for the NIST tests. Every byte of the S-box was encoded as an 8-bit binary string, all of which were concatenated to create a long string of binary digits. The NIST Statistical Test Suite (NIST) was used to test the randomness of the binary sequences that were generated. The tests verify various statistical characteristics such as frequency distribution, runs and linear complexity to identify deviations from randomness. The NIST suite calculates the probability of $p_\text {value}$ for each sequence, and if $p_\text {value}$ $\ge \lambda$ (or $p_\text {value}$ < $\lambda$ ), the sequence is considered random (or non-random), where $\lambda$ is a predefined threshold known as the significance level. For cryptographic purposes, $\lambda$ is usually set between 0.001 and 0.01^61^. The results for 14 tests including (random occurrence, random occurrence variant, etc.), which are some of the most significant for cryptographic use. These have been selected because they examine properties directly related to S-box security, such as uniformity, independence, and unpredictability. The results of the statistical randomness test of the proposed S boxes through the tool provided by NIST 800-22^61^ are listed in Table 11. The Universal Statistical Test was excluded, as it is intended for longer input sequences.

**Table 11:** The NIST results of the proposed S-boxes $\mathcal {S}_{p}$, $\mathcal {S}_{128}$, $\mathcal {S}_{224}$, $\mathcal {S}_{256}$ and $\mathcal {S}_{521}$.

S.No.	NIST test	*p*-value					Random
		$\mathcal {S}_{p}$	$\mathcal {S}_{128}$	$\mathcal {S}_{224}$	$\mathcal {S}_{256}$	$\mathcal {S}_{521}$	
1	Frequency Monobit	1.0000	1.0000	1.0000	1.0000	1.0000	$\checkmark$
2	Block Frequency	0.9936	0.2687	0.3130	0.5201	0.9624	$\checkmark$
3	Cumulative Sum	0.9977	0.5748	0.3697	0.8188	0.7375	$\checkmark$
4	FFT	0.6555	0.1443	0.5702	0.0621	0.5164	$\checkmark$
5	Runs	0.8597	0.7909	0.7570	0.8597	0.6269	$\checkmark$
6	Longest Run	1.0000	1.0000	1.0000	1.0000	1.0000	$\checkmark$
7	Non-Overlapping Template	0.8560	0.4067	0.0343	0.8501	0.8381	$\checkmark$
8	Overlapping Template	0.2957	0.4884	0.8866	0.2957	0.4884	$\checkmark$
9	Binary Matrix Rank	0.1761	0.4812	0.0852	0.7419	0.4812	$\checkmark$
10	Linear Complexity	0.1246	0.9595	0.6093	0.9595	0.0619	$\checkmark$
11	Approximate Entropy	0.8597	0.7909	0.7570	0.8597	0.6269	$\checkmark$
12	Serial test-1	0.6453	0.2262	0.5487	0.4989	0.3079	$\checkmark$
	Serial test-2	0.8750	0.1882	0.7864	0.6032	0.4633	$\checkmark$
13	Random Excursions	0.1461	0.8805	0.9362	0.8121	0.4462	$\checkmark$
14	Random Excursions Variant	0.4369	0.6221	0.4644	0.3597	0.5104	$\checkmark$

## Application of optimized S-boxes in image encryption

S-boxes used in image encryption applications are further employed to assess their strength. The process takes a plain image as input and produces an encrypted cipher image as output. The image coefficients are substituted using the proposed optimized S-boxes: $\mathcal {S}_{128}, \mathcal {S}_{224}, \mathcal {S}_{256},$ and $\mathcal {S}_{521}$. An encrypted image is generated by mapping these optimized S-boxes, each of size $16 \times 16$, onto plain text images sized $256 \times 256$.

The Majority Logic Criterion (MLC) can be utilized to evaluate the encryption capabilities of the S-boxes^62^. To evaluate the randomness of the encrypted image, MLC employs several analyses, including entropy, energy, homogeneity, contrast, correlation, and the mean of absolute deviation (MAD). Additionally, differential statistical analysis is performed to assess the Number of Pixel Change Rate (NPCR) and the Unified Average Changing Intensity (UACI). Cryptographic images can be identified by examining homogeneity and energy. A correlation test compares the plaintext and encrypted images to assess their similarity, with encryption reducing the correlation value, indicating greater distortion. Contrast is used to estimate the loss of brightness in the original image, with higher contrast scores indicating more effective encryption. MAD analysis helps quantify the quality difference between the encrypted and original images. The quality of the S-box is determined by the statistical features that emerge from the distortions introduced during the encryption process.

**Fig. 10:**
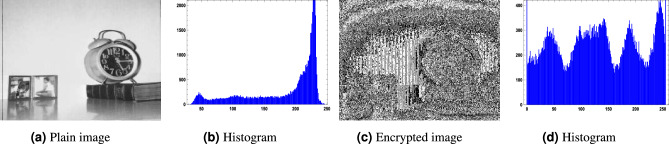
“Plain image, encrypted image and their histograms using the optimized S-box $\mathcal {S}_{128}$.

**Fig. 11:**
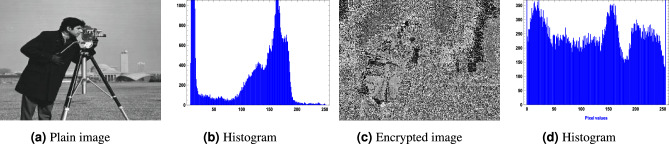
Plain image, encrypted image and their histograms using the optimized S-box $\mathcal {S}_{224}$.

**Fig. 12:**
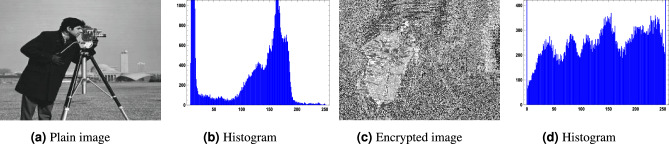
Plain image, encrypted image and their histograms using the optimized S-box $\mathcal {S}_{256}.$.

**Fig. 13:**
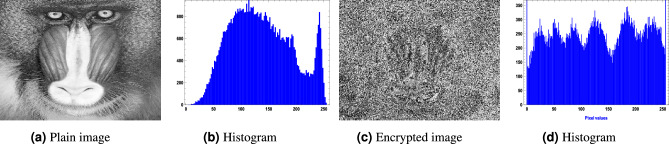
Plain image, encrypted image and their histograms using the optimized S-box $\mathcal {S}_{521}$.

**Fig. 14:**
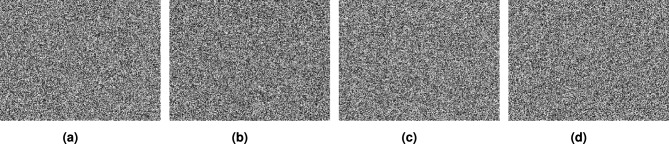
Images (**a**–**d**) show the encrypted results of the plain images generated using optimized S-boxes over multiple rounds.

**Fig. 15:**
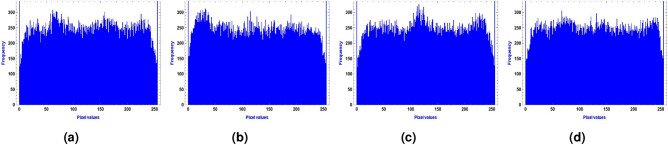
Images (**a**–**d**) show the histograms of encrypted images from Fig. 14.

For the image encryption application, we used $256 \times 256$ images of a clock, a cameraman, and a baboon, applying our generated optimized S-boxes $\mathcal {S}_{128}, \mathcal {S}_{224}, \mathcal {S}_{256},$ and $\mathcal {S}_{521}$. The encrypted images were obtained after a single round of applying the optimized S-box to evaluate the randomness of the S-box. Figures 10,  11,  12 and  13 show the results of image encryption using these optimized S boxes after applying a single round, where (a) Display the plain images; (b) provide the histograms of the plain images; (c) display the encrypted images using $\mathcal {S}_{128}$, $\mathcal {S}_{224}$, $\mathcal {S}_{256}$ and $\mathcal {S}_{521}$; and (d) present their histograms. To prevent information leakage, multiple rounds were carried out to obtain the encrypted images as shown in Fig. 14 and their corresponding histograms are shown in Fig. 15. As evident from the Figs. 14 and  15, the results are robust and effectively prevent information leakage. We analyze the encrypted images using MLC tests, comparing the results with the encryption performance of the AES S-box and the APA S-box. The results of the MLC test are shown in Table 12, while additional results for the number of pixel changes (NPCR) and the unified average change intensity (UACI) are presented in Table 13. These results demonstrate that the image encryption performance of the generated optimized S-boxes is satisfactory and consistent.

**Table 12:** The MLC results for image encryption using the S-boxes $\mathcal {S}_{128}, \mathcal {S}_{224}, \mathcal {S}_{256},$ and $\mathcal {S}_{521}.$.

Image	S-boxes	Entropy	Contrast	Correlation	Energy	Homogeneity	MAD
Clock	$\mathcal {S}_{128}$	7.9516	9.9846	0.0034	0.0158	0.3937	45.6098
	APA	7.9549	10.0489	0.0046	0.0159	0.3964	47.6342
Cameraman	$\mathcal {S}_{224}$	7.9498	10.8673	0.0013	0.0157	0.3887	42.2167
	$\mathcal {S}_{256}$	7.9543	10.7115	0.0112	0.0157	0.3858	34.6141
	APA	7.9527	9.9973	0.0046	0.0158	0.3963	42.3679
Baboon	$\mathcal {S}_{521}$	7.9573	10.7438	0.0048	0.0157	0.3881	41.3454
	AES	7.9578	10.6147	0.0013	0.0157	0.3900	47.5250

**Table 13:** NPCR and UACI results for image encryption using optimized S-boxes.

	Clock	Cameraman		Baboon
S-boxes	$\mathcal {S}_{128}$	$\mathcal {S}_{224}$	$\mathcal {S}_{256}$	$\mathcal {S}_{521}$
NPCR	99.6185	99.5270	99.7635	99.7757
UACI	33.5822	33.0100	33.8571	33.2555

## Conclusion

The task of generating S-boxes with high nonlinearity while maintaining low computational time is a significant challenge. In this work, we present a novel substitution S-box generator based on cubic Pell curves. The main idea behind this approach is to leverage the randomness inherent in the points on cubic Pell curves, using their binary representations. Our S-box generation method produces dynamic and optimized S-boxes that exhibit high nonlinearity and strong cryptographic properties. Through comprehensive cryptographic analysis, we have compared our dynamic S-box generation method with various existing methods^10,11,14,16–18,20–22,37–49,52,53,58^, including the fastest dynamic S-box generation technique^15^. Our security analysis shows that the S-boxes generated by our method demonstrate superior cryptographic strength in comparison to others. We evaluated the nonlinearity due to eight boolean functions of 10,000 S-boxes generated by our approach and found that these S-boxes possess highly nonlinear characteristics, outperforming those generated by other methods^5,10,14,15,42,44,52^. The NL due to eight boolean functions of our optimized S-boxes $\mathcal {S}_{128}$, $\mathcal {S}_{224}$, $\mathcal {S}_{256}$, and $\mathcal {S}_{521}$ is 108, which is notably higher than the NL values of other existing optimized generators^24–31,33,34^. This indicates that our optimized S-box method is capable of generating highly secure S-boxes with exceptional cryptographic properties, including nonlinearity (NL), strict avalanche criterion (SAC), linear approximation probability (LAP), bit independence criterion (BIC), differential approximation probability (DAP), and algebraic complexity (AC). The proposed S-boxes also satisfies the randomness properties when evaluated using the tests provided by NIST test suite. In addition, we tested the effectiveness of the optimized S-boxes $\mathcal {S}_{128}, \mathcal {S}_{224}, \mathcal {S}_{256},$ and $\mathcal {S}_{521}$ in the context of image encryption. The results demonstrated that the generated S-boxes contribute significantly to enhancing the security of image encryption applications. Overall, our proposed S-box generation method shows great potential for use in cryptographic systems requiring high security and robustness against various types of attacks.

Future work could focus on applying the S-box generator to real-world encryption tasks, such as securing multimedia data (images, videos) and sensitive communications. Research could also explore more efficient algorithms and mathematical approaches to improve performance without compromising security.

## Supplementary Information

Supplementary Information.

## Data Availability

Correspondence and requests for materials should be addressed to MAM. Khan.
