# Supplementary material for: An optimized substitution box generator based on cubic pell curves and its application in image encryption
Source: Sci Rep. 2026 Jan 28;16:3896. doi: 10.1038/s41598-025-28045-y (PMC12855255; doi:10.1038/s41598-025-28045-y)
Supplement: Supplementary file 1 — Supplementary Information. [file 41598_2025_28045_MOESM1_ESM.pdf]

Supplementary Material

Parameters used for experiments

bits	p, a, b, x_G, y_G	α, m, r
128 bits	p=340282366762482138434845932244680310783<br>x_0=77432292733681514512374757901724034626994555514851933961363922195693758 5<br>y_0=37225563641023208223712612458955503640790138185586007068125666572538666 8<br>z_0=17896184388572268906856039931447325499190179780178262143705391497785573 2	9, 8, 2
224 bits	p=6277101735386680763835789423207666416102355444459739541047<br>x_0=1421439799947695458721324913634646066857807643635 2001<br>y_0=6833569802308041024337659494149731268744666656885600<br>z_0=3285237703681484604434287181645818568310108059849760	9, 8, 2
256 bits	p=115792089237316195423570985008687907853269984665640564039457584007908834671663<br>x_0=27052372618466569067504367354473886314425337353954822734684141921670163322520163989691633 2<br>y_0=13005424261593103133862821846614147931455691844312337119899662765178366929496496246438750 8<br>z_0=62523558509827323144807435048277400170282120090791789615818058831314257002772152910247425	9, 8, 2
521 bits	p=686479766013060971498190079908139321726943530014330540939446345918554318339765605212<br>2559640661454554977296311391480858037121987999716643812574028291115057151<br>x_0=5217519347630008286796874534296439019811650028421025040699603560024473763339343633232265<br>90682440412402977541676954812655882202069902900051984743628214939618188581185336371530 92<br>272033554009830464704907828860981573982000<br>y_0=2508321679063323051038673155319354611152415851113235236404301959039927713917976410470900 67<br>2913277459087041310998134482107017008857597220015293998074002514059367878849534005519452625 9<br>674835866956962212218256341382190942<br>z_0=1205875287940611594278255391346679912148086784504866630233369935135039607490090749158786 32<br>9500162038470592521952438253712789241590674520487984127298894828473392149581291131268624025 5<br>123959280100136895755778273388331529	9, 8, 2
